# Research on the Construction of a Comprehensive Evaluation Model of Psychological Factors in Coal Mine Workers' Safety: Investigation and Analysis of 1,500 Miners in East China

**DOI:** 10.3389/fpubh.2022.849733

**Published:** 2022-03-03

**Authors:** Junqi Zhu, Xue Wang, Li Yang, Zhiyuan Qin, Jichao Geng, Xuesen Zhang

**Affiliations:** School of Economics and Management, Anhui University of Science and Technology, Huainan, China

**Keywords:** coal miners, comprehensive evaluation, influencing factors, investigation and analysis, safety psychology

## Abstract

With China's economic and social development entering a new era, the improvement of miners' living standards and safety production conditions in coal mine are bound to have a new impact on the safety needs of miners. In order to explore the structural changes of miners' safety demands in the new era, this research adopts the second-order confirmatory factor analysis method to investigate miners from six coal mining enterprises based on Koffka's cognitive psychology theory. Firstly, according to the interaction between the behavioral environment and the self-regulation of coal miners, six potential variables affecting miners' safety psychology, such as material satisfaction, non-skill internal causes, professionalism, emotional attribution, safety atmosphere, and organizational management, are selected. Then, each potential variable is subdivided into 3 observation variables, for a total of 18 observation variables, and a 3-tier comprehensive structural model of miners' safety psychology is constructed that takes into account both evaluation and path integration. The results showed that, affected by the interaction of various potential variables, the degree and intensity of the influence of each factor on miners' safety psychology were different. Among them, emotional attribution was the most significant factor affecting miners' safety psychology, while the influence of organizational management was slightly less important than emotional attribution. Organizational management had a positive impact on material satisfaction and non-skill internal factors. Occupational literacy, material satisfaction, and safety atmosphere had strong impacts on miners' safety psychology. But the impact of non-skill factors on miners' safety psychology was lower than other factors, which is different to previous studies on this aspect.

## Introduction

China is currently the world's largest coal producer and consumer, with coal consumption accounting for 60.4% of total energy consumption and coal resources accounting for 95% of total fossil energy in China (1, 2). China's natural resource endowment of “rich coal, poor oil, and less gas” ensures that coal will remain an important strategic resource in China for a long time to come, which also means that the safety production of coal mines and miners' safety psychological problems will be concerns for a long time. With China's economic and social development entering a new era, the main social contradictions have been transformed into the contradiction between people's growing need for a better life and unbalanced and inadequate development. This means that in the context of the new era, with the improvements in people's living standards and in coal mine safety production conditions, the working environment and demand structure of coal miners have changed, and the influencing factors and the role of factors in past research on miners' safety psychology will also change quietly with the changes in environment and conditions. The uncertainty in miners' safety psychological factors will gradually become more prominent. So then, how will the factors affecting the safety psychology of coal miners change? What factors will continue to play a role? To what extent will they play a role? Are there interactions among these factors? Are there new factors? What are the new requirements for the safety production management of coal mines? This paper, based on the current needs of the coal industry in China, conducts a comprehensive psychological evaluation of coal miners' safety from the perspective of cognitive psychology, relying on K. Koffka's cognitive psychology theory, on the basis of a literature review and according to the interaction of the behavioral environment and self-regulation of coal miners in the new era. Six potential variables affecting miners' safety psychology—material satisfaction, non-skill internal causes, occupational literacy, emotional attribution, safety atmosphere, and organizational management—are selected. Each potential variable is subdivided into 3 observation variables, a comprehensive evaluation system for miners' safety psychology with a 3-layered structure is constructed, and its structure is analyzed using 18 observation variables. The second-order confirmatory factor analysis method is used to construct a comprehensive structural model of miners' safety psychology that takes into account both evaluation and path integration. The relationships and interactions among the factors are comprehensively analyzed, and various derivative factors and structural characteristics affecting miners' safety psychology in the new era are explored.

The significance of this study is to clearly grasp the key factors affecting the safety psychology of miners, and then to clarify the effect degree and mutual relationship between the key factors. According to the psychological needs of miners and the mutual relationship between the factors, the managers of coal mine enterprises can adjust the management mode and incentive mode in time, and provide more targeted safety services for miner to prevent the occurrence of safety accidents, which can effectively guarantee the physical and mental safety of miners and the safety of coal mine production.

## Literature Review and Model Construction

Cognitive psychology holds that the core of human psychology and behavior is cognitive problems (3, 4). Human behavior depends on the interaction between their behavioral environment and self-regulation (2). Individual behavior is not an isolated and simple response to external stimuli, but is made through the integration of psychophysical fields including environment and self, especially cognitive activities (3). Behavior occurs through the behavioral environment (5). Self is a personality system closely related to psychological activities, such as needs, hopes, cognition, emotions, and attitudes (6). Specifically for coal miners, their safety psychology is based on the interaction of the behavioral environment and self-cognition. Therefore, the premise and foundation of accurately measuring and quantifying miners' safety psychology is to clarify the influencing conditions and key factors affecting miners' safety psychology. Based on the literature review and starting from the environment and self-regulation of the coal mining industry in the new era, we summarize six potential variables affecting miners' safety psychology including material satisfaction, non-skill internal causes, professionalism, emotional attribution, safety atmosphere, and organizational management. These factors are brought about by the improvement of production conditions and living conditions. Among them, material satisfaction, non-skill internal causes, professionalism, and emotional attribution brought about by the improvement of production conditions and living conditions are self-regulating variables, while safety atmosphere and organizational management are important environmental variables.

### Material Satisfaction

Family economic environment and material security are important factors affecting people's choice of the miner occupation (7). Firstly, the important reason why people choose the miner occupation is their need for social survival. Salary and welfare are the primary motives of miners' work. The effective satisfaction of salary and welfare will generate direct power for the safe and stable operation of miners. In addition, equipment conditions are “hard conditions” that affect miners' safety psychology. From the reports on various major accidents, we can understand that the imperfect or unreasonable operation of equipment will bring about hidden dangers in coal mines. Relevant studies show that the level of mechanization of coal mining has the greatest impact on the number of deaths in coal mine accidents (8, 9).

### Non-skill Internal Causes

Non-skill internal causes are such factors as the physiological functions, self-efficacy, and emotional regulation that coal miners are born with and can be regulated through acquired training. Studies found that the poor mental state of miners is an important factor leading to their unsafe behavior (7, 10–13). Human physiological activities are affected by many factors. The health status and mental state of coal industry practitioners will affect their decision-making and actions, resulting in illegal commands and operations, thus leading to safety accidents. In addition, self-efficacy is a subjective assessment of a person's ability to complete certain aspects of his/her work. Self-efficacy influences individual behavior through emotions, awareness, and attitude, and then influences safety behavior through safety awareness, risk perception, and safety attitude. Emotions also have an important impact on the safety of miners. The mechanism of emotional fluctuations causing work errors is as follows: Abnormal emotions will interfere with the brain's information input-processing-response output process, so as to reduce the reliability of work and even lead to mistakes. Emotions have a positive regulatory effect on the relationship between safety self-efficacy and safety awareness (3, 6, 14, 15). Impetuous, flirtatious, unresponsive, and indifferent attitudes caused by negative emotions easily lead to idleness, misjudgment, illegal operations, etc.

### Professionalism

Professional is the normative operational ability and behavior performance of coal miners in operational activities, including professional awareness, responsibility consciousness, safety operational ability, etc. (16, 17). Miners' professional awareness affects their professional accomplishment. Due to the limitations of certain historical conditions, uneven professional awareness by miners will inevitably be reflected in their standard operational ability. The level of miners' ability is related to the quality of equipment operation and the frequency of safety accidents. At the same time, the sense of responsibility has an important influence on the safety psychology of miners. Responsibility consciousness is manifested in the responsibility for oneself and one's surrounding environment, and the choice to maximize benefits. Out of concern for their own safety, miners are prone to have a “morning well” mentality of early safety in the morning. They pursue the working methods of saving labor, reducing time, and avoiding heavy work. Moreover, miners' safety knowledge, consciousness, and habits may lead to improper safety operation, negligence, and numbness, which directly or indirectly affect the safety of coal mine production (10–13, 18).

### Emotional Attribution

The emotional attribution, also known as the sense of belonging or the perception of affiliation, refers to an internal relationship between an individual and their subordinate group. It is the psychological expression of a certain individual's delimitation, identification, and maintenance of a special group and their subordinate relationship (7). First, a group relationship is manifested as the activity and identity of individuals in group interactions. Group dynamics theory (19) points out that group members all want to be accepted by other members of the group, and the pressure of disagreement will lead to the opposite behavior by group members against their own wishes. Due to the limitation of shift scheduling and working hours, communication among miners is conducive to alleviating mental and physical pressures. Group value orientation and behavior choice also make miners' safety psychology fluctuate. Secondly, family relationships are the main relationships among miners. Long-term separation or loneliness will lead to loneliness and dependence for miners, which will affect their psychological mood and safety psychology. Finally, the social support obtained by miners is positively correlated with their mental status (20). Coal miners' low social status, lack of due respect socially, and low sense of professional identity result in discontent and retaliation, leading to dangerous behavior (21).

### Organizational Management

Behavioral norms, information exchange, and penalties in organizational management are the most obvious responses to the safety operation of miners (7). To a certain extent, the management's awareness of safety determines the safety quality of employees in coal mine enterprises. The loopholes in the management process mainly lie in the lack of formulation or improvement of operation rules or the lack of attention to operation rules and the implementation degree. At the same time, the timeliness and reality of information flow have a direct impact on employee safety participation behavior. In addition, there is a significant negative correlation between violation penalties and unsafe behavioral intentions and behaviors (22). The rewarding or punitive incentives imposed by mining enterprises on their employees will have a direct and significant impact on their safety compliance behavior (23). That is to say, reasonable formulation of various management mechanisms and reward and punishment systems for violations of regulations and penalties is conducive to improving the behavioral norms of the mining industry.

### Safety Atmosphere

A safety atmosphere is embodied in coal miners' understanding and internalization of safety policy, accident experience, and organizational behavior (7, 24, 25). First of all, a country's overall environmental and safety policy guidance will have a great impact on coal mine safety production. With states attaching importance to the safety work of the coal mine industry, safety investment has been continuously improved, and the overall safety situation of the coal mine industry has gradually improved. Secondly, a series of physiological, emotional, cognitive, and behavioral stress reactions (26) will occur after a period of personal psychological influence, paying attention to coal mine safety accidents, or personal experience. Miners' normal working ability will be disturbed, which will increase the probability of misoperation and unsafe behaviors (27); this will have a lasting impact on group safety awareness and the formation of a safety culture. Finally, the behavior and organizational behavior of miners will affect individual behavioral choices. Once a safety culture is formed, it will exert a profound and lasting influence on miners' safety behavior and safety psychology (28).

In summary, the following 6 potential variables and 18 observational variables affecting miners' safety psychology were established in this study (Table 1).

**Table 1:** Potential and observed variables table.

**Potential variable**	**Observed variable**	**Potential variable**	**Observed variable**
Professionalism	Professional degree	Emotional attribution	Family relations
	Sense of responsibility		Professional identity
	Safety consciousness		Group relationship
Non-skill internal causes	Emotional regulation	Safety atmosphere	Accident experience
	Physiological state		Security culture
	Self-efficacy		Security policy
Material satisfaction	Institutional guarantee	Organizational management	Code of conduct
	Salary and welfare		Information change
	Equipment conditions		Violation punishment

Previous research on miners' safety psychology mostly focused on single factor analysis or normalization of miners' safety psychology, and most relevant studies are carried out from the perspective of enterprise management. For example, Gao believes that the characteristics of miners themselves, including their physiological and psychological states during production, will affect the accuracy of their judgments and behaviors—in particular, miners' bad psychological factors will lead to violations (29). Liu and others believe that “team safety construction” and “leading group” are more recognized, but we still need to pay attention to the “safety management departments” and “laws and policies” factors, and also need to further improve the “work responsibility” and “psychological quality” factors (11). Few studies have constructed a multi-factor comprehensive evaluation model for empirical measurement from the perspective of interaction between internal factors and external behavior environment.

**Figure 1:**
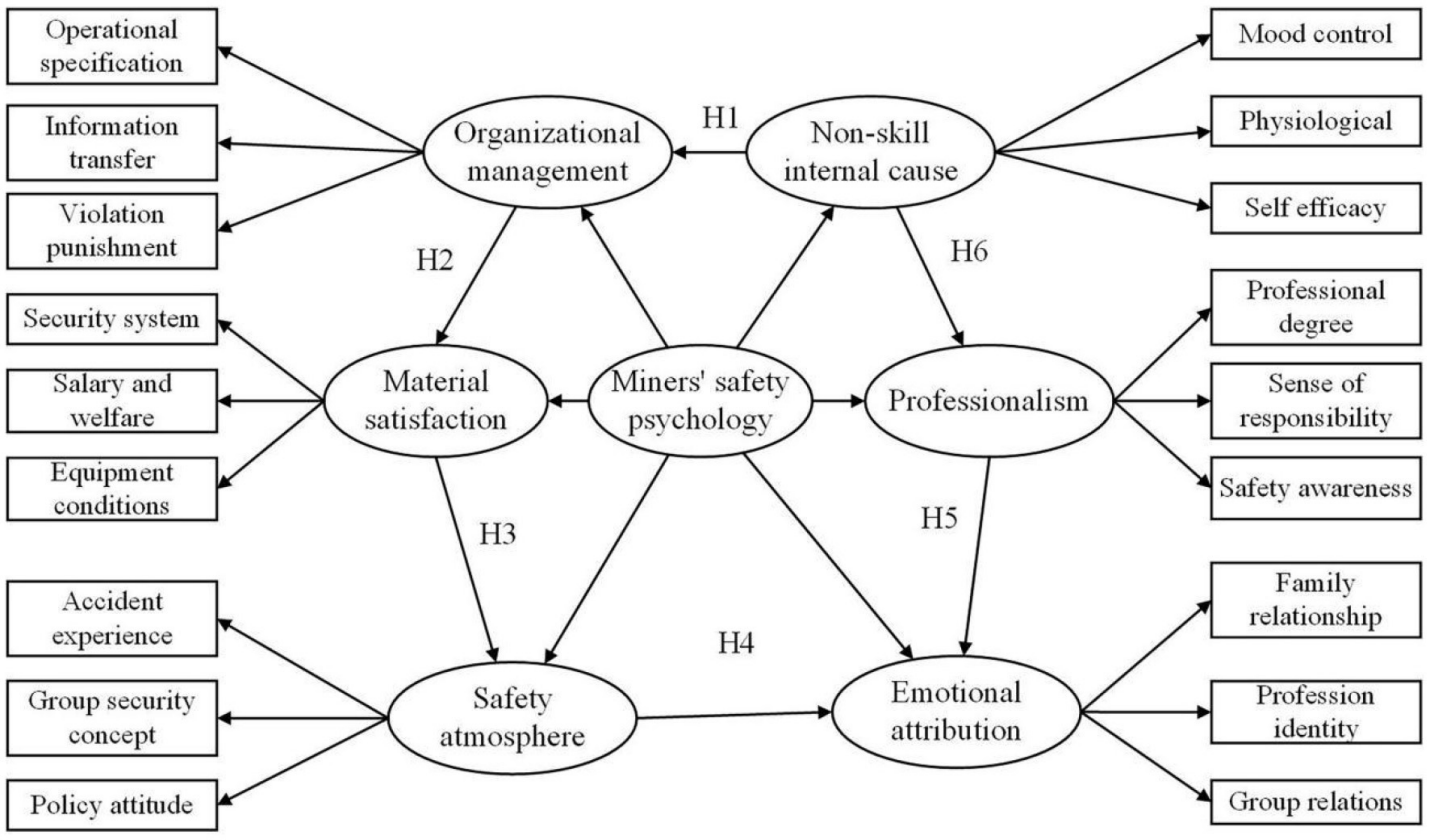
Comprehensive model of three-level structure.

The innovation of this study lies in that six potential variables affecting the safety psychology of coal miners are summarized based on the cognitive psychology theory of K. Koffka, the interaction between the behavioral environment and self-regulation of coal miners in the new era, and previous literatures. This paper further uses second-order confirmatory factor analysis method, analysis the relationship between various factors and interactions. Construct a comprehensive three-level structural model of miners' safety psychology which takes account of both evaluation and path integration (Figure 1), and more targeted exploration of the new era of miners' safety psychology of various derivative factors and structural characteristics.

## Sampling Design and Questionnaire Analysis

Based on the above 6 potential variables and 18 observation variables that affect miners' safety psychology, the questionnaire, sampling design, and model optimization were carried out in this study.

### Sampling Design

This study combines random sampling with Probability-Proportional-to-Size Sampling. Based on the distribution of coal mines in A and B cities, six coal mines were selected randomly. Then, according to the total number of miners in six coal mines and the arrangement of underground work, a certain number of groups were selected according to the Probability-Proportional-to-Size Sampling method, and then random miners were surveyed in the selected groups.

The formula based on sample size was *n* = Z^2*^[P^*^(1-P)/ E^2^]. If the confidence level was 95% (*Z* = 1.96), the error value was *E* = 3%, and the probability value was *P* = 0.5; so, a sample size of *n* = 1,067 could be obtained. Therefore, our sample size needed to be at least 1,067. Considering the scale of the enterprises we investigated and in order to minimize sampling errors, with the cooperation of coal mining enterprises, we appropriately expanded the sample size and finally determined 1,500 copies. The total number of people in the 6 coal mines was about 54,000. Thus, the sample size of 1,500 coal miners did not exceed 5% of the total. The sample size did not need be adjusted. Considering that all six coal mining units adopt district, queue, and group scheduling, we adopted phased sampling. We used district as the primary sampling unit (PSU). In the 46 working areas of the 6 mining enterprises, 20 were selected; then, 5 teams were drawn in each working area by drawing lots, and 5 working groups were selected for each team. Finally, 3 people were randomly selected as samples in the group, totaling 1,500 people (Table 2).

**Table 2:** Group distribution of coal mine workers in two cities: A and B.

**Serial number**	**Unit name**	**Working area code**	**PSU scale**	**PSU** **accumulative scale**	**Sampling range**	**Selected number**
1	ZJ coal mine	D-1	1,000	1,000	(1–1,000)	
2		D-2	1,200	2,200	(1,001–2,020)	1,123
3		D-3	1,200	3,400	(2,021–3,400)	
4		D-4	1,000	4,400	(3,401–4,400)	3,832
5		A-1	500	4,900	(4,401–4,900)	
6		A-2	700	5,600	(4,901–5,600)	
7		C-1	1,000	6,600	(5,601–6,600)	6,541
8		C-2	750	7,350	(6,601–7,350)	
9		C-3	1,200	8,550	(7,351–8,550)	
10		B-1	1150	9700	(8,551–9,700)	9,250
11	GQ coal mine	B1-S	1,100	10,800	(9,701–10,800)	
12		B1-W	1,300	12,100	(10,801–12100)	11,959
13		B1-N	1,350	13,450	(12,101–13,450)	
14		B2-E	1,350	14,800	(13,451–14,800)	14,668
15		B2-N	1,350	16,150	(14,801–16,150)	
16		B2-S	1,300	17,450	(16,151–17,450)	17,377
17		B3-S	1300	18,750	(17,451–18,750)	
18		B3-N	1,300	20,050	(18,751–20,050)	
19	DT coal mine	W-1	1,120	21,170	(20,051–21,170)	20,086
20		W-2	1,120	22,290	(21,171–22,290)	
21		N-1	1,120	23,410	(22,291–23,410)	22,795
22		N-2	1,110	24,520	(23,411–24,520)	
23		N-3	1,110	25,630	(24,521–25,630)	
24	SH coal mine	SW-1	1,110	26,740	(25,631–26,740)	25,504
25		SW-2	1,133	27,873	(26,741–27,873)	
26		SE-1	1,200	29,073	(27,874–29,073)	28,213
27		SE-2	1,200	30,273	(29,074–30,273)	
28		NW-1	1,200	31,473	(30274–31473)	30,922
29		NW-2	1,133	32,606	(31,474–32,606)	
30		NW-3	1,130	33,736	(32,607–33,736)	33,631
31		N-1	1,130	34,866	(33,737–34,866)	
32	PYD coal mine	A-1	1,900	36,766	(34,867–36,766)	36,340
33		A-2	1,875	38,641	(36,767–38,641)	
34		B-1	1,893	40,534	(38,642–40,534)	39,049
35		B-2	1,285	41,819	(40,535–41,819)	41,758
36		B-3	1,304	43,123	(41,820–43,123)	
37		C-1	1,366	44,489	(43,124–44,489)	44,467
38		C-2	1,352	45,841	(44,490–45,841)	
39	PS coal mine	PS1	966	46,807	(45,842–46,807)	
40		PS2	973	47,780	(46,808–47,780)	47,176
41		PS3	960	48,740	(47,781–48,840)	
42		PS4	1,015	49,755	(48,841–49,755)	
43		PS5	983	50,738	(49,756–50,738)	49,885
44		PS6	1,124	51,862	(50,739–51,862)	
45		PS7	1,176	53,038	(51,863–53038)	52,594
46		PS8	1,142	54,180	(53,039–54,180)	
		Total	54,180			

The first step was to determine the arrangement of personnel in the six coal mines. The scale of workspace personnel meeting the survey requirements was arranged and the sampling range of each workspace was given.

The second step was to extract the working areas from PPS sampling. First, the interval sampling of *K* = 54,180/20 = 2,709 was calculated. Using a random number table, a random starting point of 1,123 was given in the range of 1~K. From the total population of 54,180 people, 20 PSU were extracted. According to the principle of equidistant sampling, the second one was 1,123 + 2,709 = 3,832, and so on; in this way, the sampling frame was obtained (Table 3).

**Table 3:** Sampling frame distribution table.

**Sampling frame**	**Working area**
ZJ coal mine	D-2, D-4, C-1, B-1
GQ coal mine	B1-W, B2-E, B2-S
DT coal mine	W-1, N-1
SH coal mine	SW-1, SE-1, NW-1, NW-3
PYD coal mine	A-1, B-1, B-2, C-1
PS coal mine	PS-2, PS-5, PS-7

The third step was to select five teams by drawing lots in each selected working area.

In the fourth step, starting with 100 teams, the team numbers were found using the random number table method, and 5 teams were extracted.

In the fifth step, in each selected group, three people were surveyed by drawing lots. We drew lots according to the specific number of people in the group, found the workers selected by seat, and then conducted a questionnaire survey.

### Questionnaire Issuance

To validate the model and hypothesis, the research team conducted a 9-month empirical study (1.2018–9.2018). Six potential variables and 18 observation variables were measured using the multi-index measurement method. Each measurement statement was measured with a seven-step Likert scale to measure the respondents' different attitudes or tendencies. In the scale, 1 = always, 2 = often, 3 = sometimes, 4 = unclear, 5= occasionally, 6= very little, and 7 = never. A total of 1,500 questionnaires were distributed throughout the process. The data from the questionnaires were manually checked to verify whether the filling was complete, whether the contents were logical, and whether there were contradictions between the front and back. After adjusting or eliminating questionnaires, 1,170 questionnaires were finally identified as valid samples, which accounted for 78% of the total sample. The investigation was divided into two parts: basic information and investigation content. Among them, the basic information mainly included sex, age, marital status, salary level, length of service, and the city where the household registration is located (Table 4). The contents of the survey were carried out from six aspects: miners' professional literacy, non-skill internal causes, safety atmosphere, organizational management, material needs, and emotional attribution. The survey was conducted in the form of face-to-face oral inquiries and self-filled questionnaires by the respondents. The investigators had direct contact with the respondents. The investigators used pre-designed questionnaires to learn the miners' psychological safety states, and then collected the questionnaires on the spot to ensure a good recovery rate. For the oral inquiries, we questioned the miners according to the content of the questionnaire itself. Because some of the interviewees had insufficient knowledge or understanding, our investigators filled in the questionnaire according to the respondents' answers. Self-filled questionnaires were also distributed by the investigators on the spot, and completed by the miners themselves with explanation and guidance.

**Table 4:** Demographic characteristics of survey samples.

**Variable**	**Definition of variable**	**Percentage%**	**Variable**	**Definition of variable**	**Percentage%**
Gender	Male	86.1	Marital status	Married	91.2
	Female	13.9		Unmarried	8.8
Education level	Primary school and below	5.1	Age	Under 25	4.1
	Middle school	16.3		26–35	27.9
	High school or Secondary specialized school	40.8		36–45	37.1
	Undergraduate university	36.1		46–55	29.6
	Master's degree or above	1.7		Over 55	1.4
Pay level	<2,000	2.7	Working years	<1 year	3.4
	2,000–2,500	7.1		1–2	1.4
	2,500–3,000	17.3		2–3	2.4
	3,000–3,500	24.5		3–4	4.4
	More than 3,500	48.3		More than 4 years	88.4
Domicile	Countryside	38.4	Nature of work		
	Town	16.3		Down hole workers	49.0
	County town	15.3		Management personnel	31.6
	Small and medium-sized cities	28.2		Security personnel	19.4
	Big city	1.7			

### Questionnaire Analysis

We used SPSS 20.0 software to analyze the data. The goal was to test the difference between the items of the questionnaire or the homogeneity of the items. The results of the analysis could be used to identify whether there were unreasonable items and whether the questionnaire as a whole had good reliability or not.

#### Project Analysis

The project analysis was intended to confirm the suitability of the indicators designed in the questionnaire—that is, whether there were significant differences in the evaluation of indicators among different samples, and then the necessary reduction of the indicators could be done. Generally, extreme analysis is used in project analysis. Firstly, according to the comprehensive score of this sample, the first 5% sample was defined as the high group, and the last 5% sample was defined as the low group. Then, an independent sample test was carried out for the high and low groups. The scores and total scores of the 18 indicators were tested by extreme group value, and the *P* < 0.05. This shows that there was a significant difference between the different samples when it came to the safety psychological assessment of the miners.

#### Reliability Analysis

Reliability refers to the consistency of the results of repeated measurements of the same object by the same method. The Cronbach's alpha test is generally used to test reliability, which is believed to be very credible when the coefficient is above 0.7. The Cronbach coefficients of the six potential variables passed the test criteria (Table 5), and the alpha coefficient of the full scale reliability test was 0.853 > 0.8 (Table 6), which showed that the reliability of each variable was acceptable.

**Table 5:** Reliability test results of latent variables.

**Name of variable**	**Number of items**	**Cronbach's alpha**
Professionalism	5	0.797
Non-skill internal causes	5	0.822
Material satisfaction	6	0.718
Emotional attribution	6	0.773
Safety atmosphere	4	0.862
Organizational management	5	0.738

**Table 6:** Reliability test results of the whole scale.

**Cronbach's alpha**	**Sample number**	**Number of terms**
0.853	1,170	31

#### Validity Analysis

Validity analysis is generally achieved by factor analysis; that is, a suitable factor analysis indicates that the indicators have significant structural characteristics. The questionnaire items were based on a scale obtained from the reference literature, not from the subjective design. The item design was revised at a later stage. The research items could really express the concept of the research variables. According to (30), the closer the KMO value is to 1, the greater the sum of squares of simple correlation coefficients between all variables is, the more suitable for factor analysis. Factor analysis is best when KMO > 0.9, acceptable when KMO > 0.7, and unsuitable when KMO > 0.5. It can be seen from Table 7 that the KMO value was 0.828, and factor analysis was appropriate. Bartlett's sphericity test results showed that the approximate chi-square value was 6708.790 and the significance was *p* = 0.000 < 0.05, which indicated that the data from the normal distribution were also suitable for factor analysis.

**Table 7:** Inspection of KMO and Bartlett.

**Sampling adequacy of Kaiser-Meyer-Olkin**	**0.828**	
Sphericity test of Bartlett	Approximate chi-square	6708.790
	Df	465
	Sig.	0.000

## Data Fitting and Model Optimization

### Evaluation Model

**Figure 2:**
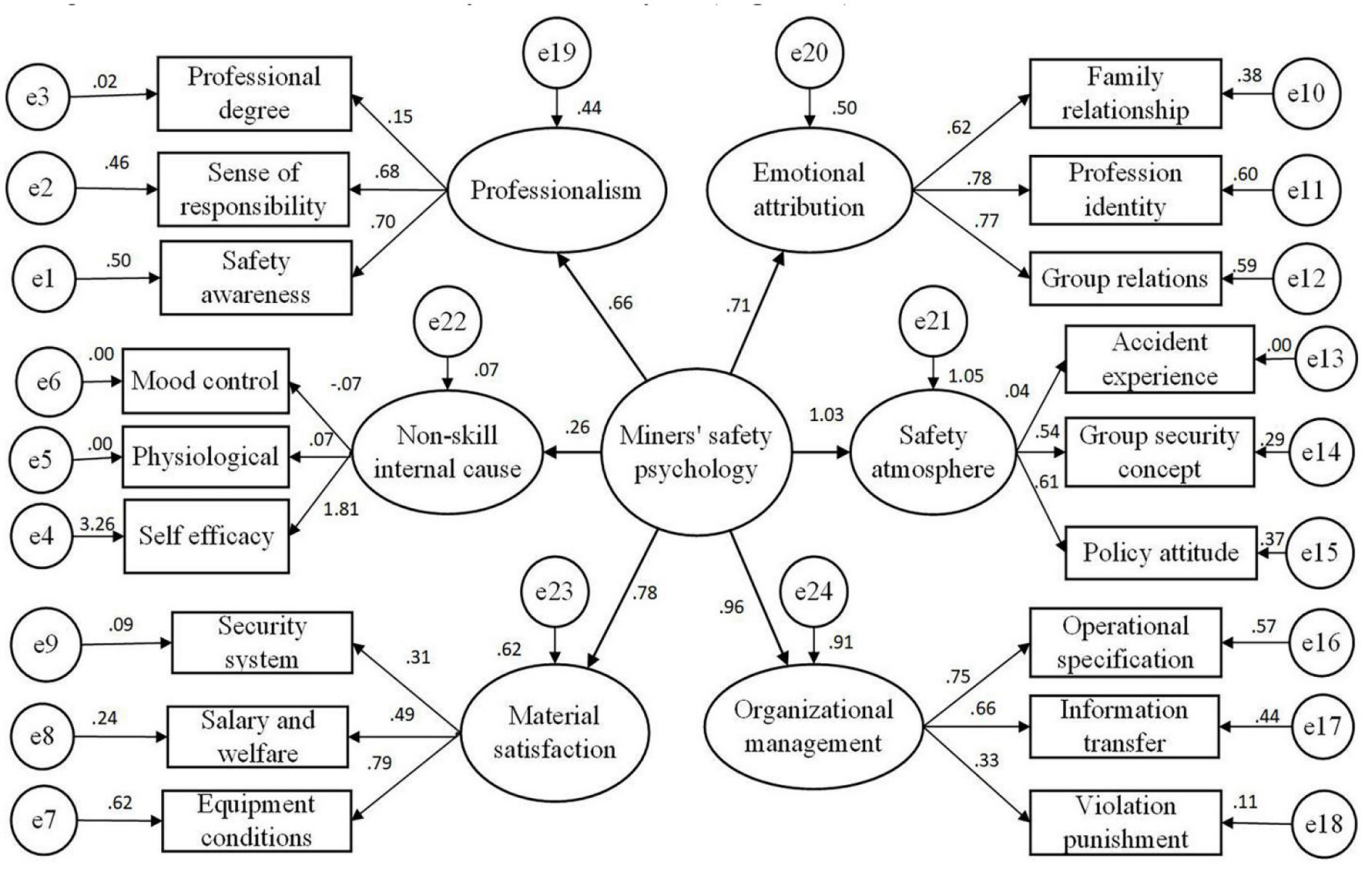
Three layers of safety psychology for miners.

In this study, the basic data of 6 potential variables and 18 observation variables were standardized and introduced into the miners' safety psychological evaluation system with a 3-tier structure. Factor loads of 18 observational variables on 6 potential variables could be obtained through second-order confirmatory factor analysis (Figure 2).

The model operation showed that the evaluation model with a simple three-tier structure was not ideal, and the factor load of the safety atmosphere on miners' safety psychology and self-efficacy on non-skill internal causes led to an “irregular estimation.” The absolute test, relative test, and coefficient test of the model were not ideal. The chi-square value/DOF value was 35.478 > 5, goodness-of-fit index (GFI) (0.810), comparative fit index (CFI) (0.641), normed fit index (NFI) (0.635), Tucker-Lewis index (TLI) (0.574)—all lower than the standard value of 0.9, and root mean square error of approximation (RMSEA) (0.121) was higher than the standard value of 0.1 (Table 8). The six potential variables of occupational literacy, non-skill internal factors, material satisfaction, mental needs, safety atmosphere, and organizational management were in conflict with the actual situation. For example, the importance of demand satisfaction was only third, which is in line with the current safeguard system of the miners known in the actual survey process. Salaries, benefits, and other reactions did not match. We believe that there may have been multiple path relationships among the six potential variables, and the potential variables had an obvious correlation, which led to duplication of the evaluation information, thus distorting the importance of the potential variables. Therefore, on the basis of the three-tier structural evaluation model, we introduced the path relationships of the six potential variables into the structural equation to achieve the dual goals of miners' safety psychological analysis and path analysis.

**Table 8:** Factor fitness test.

**Statistical test quantity**	**Suitable standard or critical value**	**Model**
*x*^2^ value	*P* > 0.05 (under significant level)	4576.630 (*p* = 0.000 <0.05)
*x*^2^ value of degree of freedom ratio	<2.00	35.478
RMSEA	<0.10	0.121
NFI	>0.90	0.635
TLI	>0.90	0.574
GFI	>0.90	0.810
CFI	>0.90	0.641
PNFI	>0.50	0.535
PGFI	>0.50	0.611

### Model Optimization

#### Initial Model

**Figure 3:**
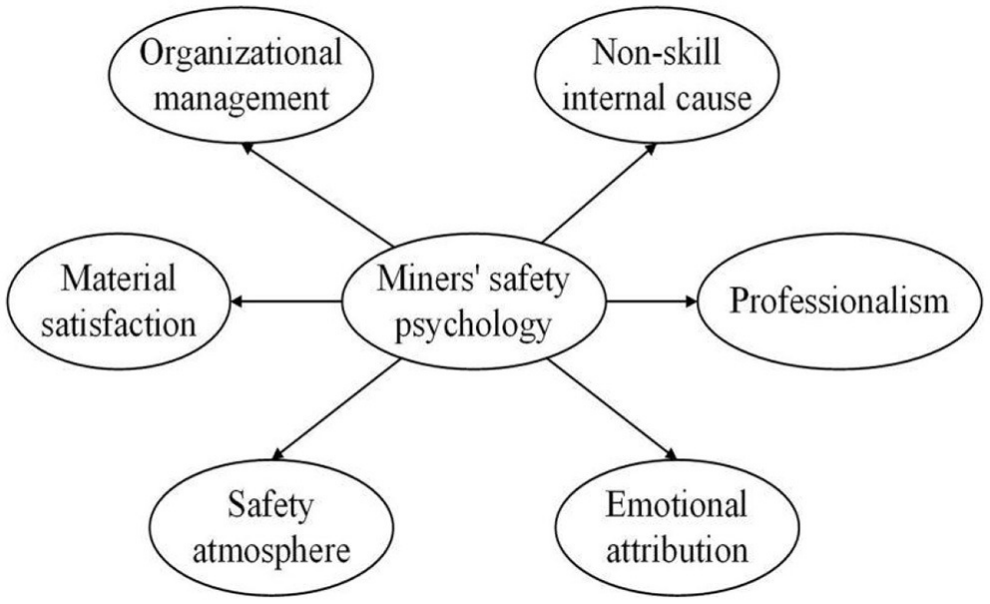
Structural equation diagram of latent variables in evaluation model.

Based on the six potential variables of the safety psychology of miners, the structural equation of the latent variable evaluation model was established (Figure 3).

#### Path Model

Previous research shows that the standardization of the management level will improve the efficiency of the organization (23, 31). The organization efficiency of ascension will further improve the welfare of the individual level and hard guarantee conditions, so as to promote individual security and create a relaxed and harmonious atmosphere (8, 9, 25). Good atmosphere of safety can alleviate the pressure of the job, increase an individual's identity to organization, and enhance individual's sense of belonging to the organization (20, 21). In addition, the skills and professional level of employees are also important aspects that affect the emotional dependence and sense of belonging in the organization. Better skills mean easier access to the trust of the organization, better acceptance by the organization, and continuous strengthening of individual organizational identity (16, 17, 19). Meanwhile, individual skills and professional level are closely related to individual work self-efficacy, emotion, mental state and other non-skilled internal factors (10–12). Good physical and mental state will enable individuals to better maintain professional quality in operations and be more standardized, professional, sober and responsible in specific operations, which having an important impact on the standardized management level of organizations (7, 22, 32). Therefore, it can be seen that there is a mediation effect or transfer effect among the six potential variables to some extent.

Based on the interrelationships between the six potential variables, the following assumptions were made:

H1: “Organizational management” has a significant impact on “material satisfaction.”

H2: “Material satisfaction” has a significant impact on the “Safety atmosphere.”

H3: “Safety atmosphere” has a significant impact on “emotional affiliation.”

H4: “Professionalism” has a significant impact on “emotional affiliation.”

H5: “Non-skill internal causes” have a significant impact on “professionalism.”

H6: “Non-skill internal causes” has a significant impact on “Organizational management.”

**Figure 4:**
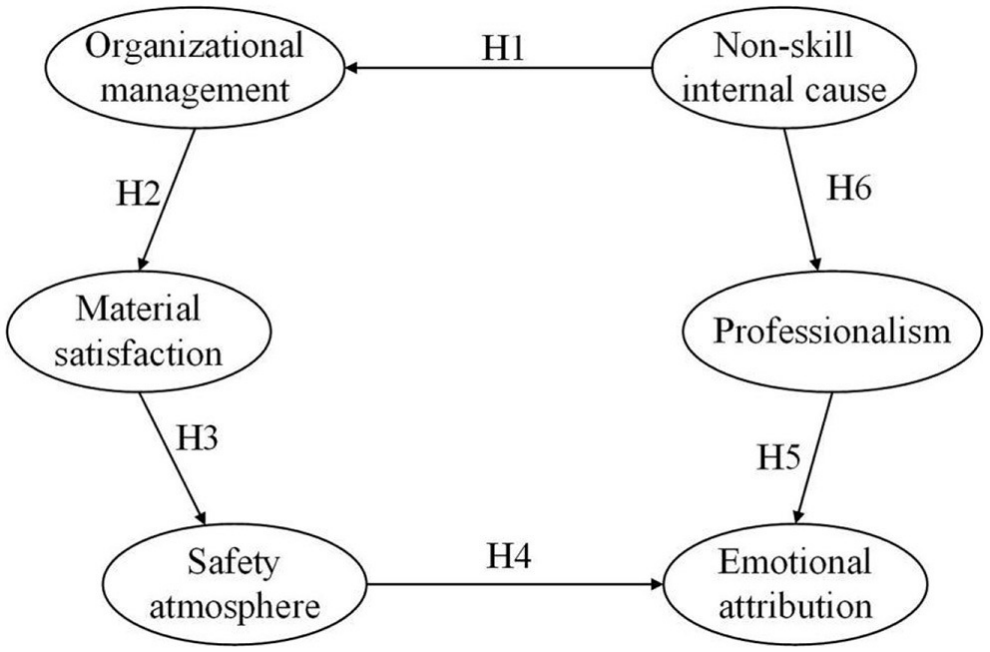
Structural equation map of path model.

Based on the above assumptions, the six structural equations of the latent variable path models were established (Figure 4).

#### Comprehensive Model

**Figure 5:**
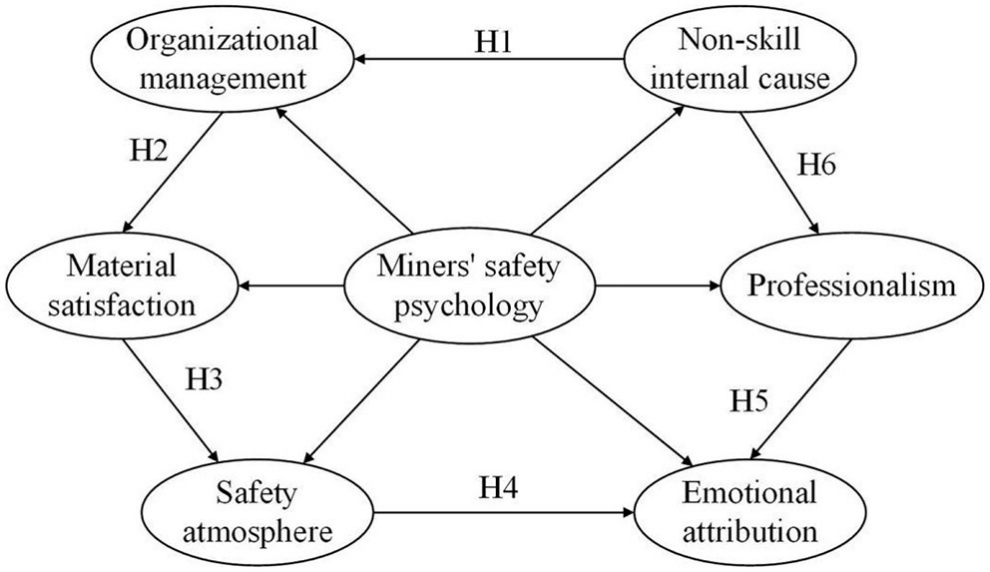
Structural equation diagram of comprehensive model.

The path model was introduced into the evaluation model, and the structural equation of the comprehensive model was constructed (Figure 5).

## Comprehensive Model Checking and Evaluation

### Violation Estimate

Before testing the fitness of the comprehensive evaluation model, it was necessary to test the “violation estimation” of the model to verify whether the fitting coefficient of the model exceeded the acceptable range. In general, there were two sub-items in the “offense estimate” inspection item: (1) whether there was a negative error variance and (2) whether the standardization coefficient exceeded or approached 1 (usually with a threshold of 0.95). The examination (Table 9) showed that the minimum error variance in the model was 0.013, and there was no negative error variance. The absolute value of the standardized coefficient in the model was between 0.015 and 0.932, not exceeding 0.95. Therefore, there was no violation estimation in the model, and the overall model fit test could be carried out.

**Table 9:** Variance and normalization coefficient table of model.

	**Estimate**		**Estimate**
Organizational management←Miner safety psychology	0.932	e19	0.036
Material satisfaction←Miner's safety psychology	0.631	e20	0.051
Non-skill internal cause←Miner's safety psychology	0.290	e21	0.022
Non-skill internal cause←Organizational management	0.831	e22	0.013
Material satisfaction←Organizational management	0.437	e23	0.057
Professional accomplishments←Miner's safety psychology	0.587	e24	0.158
Safety atmosphere←Miner's safety psychology	0.694	e1	0.473
Safety atmosphere←Material satisfaction	0.582	e2	1.990
Professional accomplishments←Non-skill internal cause	0.368	e3	0.967
Emotional attribution←Miner's safety psychology	0.894	e4	0.384
Emotional attribution←Safety atmosphere	0.449	e5	1.048
Emotional attribution←Professional accomplishments	0.608	e6	1.494
Equipment condition←Material satisfaction	0.652	e7	2.480
Salary and welfare←Material satisfaction	0.382	e8	0.716
System guarantee←Material satisfaction	0.242	e9	0.208
Operation specification←Organizational management	0.709	e10	0.844
Information interchange←Organizational management	0.663	e11	0.428
Violation punishment←Organizational management	0.274	e12	0.816
Emotion regulation←Non-skill internal cause	0.162	e13	0.454
Physiological status←Non-skill internal cause	−0.033	e14	0.788
Self-efficacy←Non-skill internal cause	−0.849	e15	0.508
Professional degree←Professional accomplishment	0.178	e16	0.953
Responsibility consciousness←Professional accomplishment	0.724	e17	0.710
Safety consciousness←Professional accomplishment	0.656	e18	1.212
Group relationships←Emotional attribution	0.755	Policy attitude←Safety atmosphere	0.538
Career identity←Emotional attribution	0.791	Safety culture←Safety atmosphere	0.490
Family relations←Emotional attribution	0.623	Accident experience ←Safety atmosphere	0.015

### Goodness of Fit Test

In the evaluation of goodness-of-fit, the higher the goodness-of-fit, the more useful the model and the more meaningful the parameter estimation. Chi-square statistics are often used to test the fitness of a model. When the model is completely suitable for the data, the difference value is 0. If the model is not suitable for the data, the difference value is infinite. So, the smaller the general requirement, the better the situation. The chi square value P is the probability of return using the difference value and the degree of freedom. With *P* > 0.05, it can be concluded that the model and data fit appropriately; with *P* < 0.05, it can be concluded that the model and data fit inappropriately. The chi-square value of this model was 201.352, and the return probability was 0.733 (Table 10). Chi-square statistics are easily affected by sample size, so besides chi-square statistics, other fitness indexes need to be consulted at the same time. The commonly used statistics for the goodness of fit test are as follows: root mean square error of approximation (RMSEA), goodness-of-fit index (GFI), adjusted goodness-of-fit index (AGFI), normed fit index (NFI), Tacker-Lewis index (TLI), incremental fit index (IFI), comparative fit index (CFI), parsimony-adjusted NFI (PNFI), and parsimony goodness-of-fit index (PGFI) (Table 11). According to the data in the table, the indicators of each statistic were up to the standard. The value-added fitting test showed that the fitting effect of the model was satisfactory. At the same time, it was necessary to test the model coefficients of the comprehensive structural model; that is, to test the non-standardized coefficients of the structural equation (Table 12). The results showed that the coefficient P of the model was <0.05, and all paths passed the test.

**Table 10:** Chi square test results.

**Statistical test quantity**	**Suitable standard or critical value**	**Model**
*x*^2^ value	*P* > 0.05 (Under significant level)	201.352 (*p* = 0.733 > 0.05)
*x*^2^ value of degree of freedom ratio	<2.00	1.637

**Table 11:** Model fitness factor test table.

**Statistical test quantity**	**Suitable standard or critical value**	**Model**
RMSEA	<0.10	0.084
NFI	>0.90	0.901
TLI	>0.90	0.908
GFI	>0.90	0.925
CFI	>0.90	0.684
PNFI	>0.50	0.560
PGFI	>0.50	0.623

**Table 12:** Model non-standardized coefficient test results.

	**Estimate**	***P***		**Estimate**	***P***
Organizational management ← safety psychology	1.555	***	Emotional attribution←Professional accomplishments	−2.843	***
Material satisfaction←Safety psychology	1.000		Information interchange←Material satisfaction	1.000	
Non-skill internal cause←Safety psychology	−2.056	***	Punishment of violations←material satisfaction	0.985	***
Non-skill internal cause←Organizational management	0.985	***	Emotional regulation←material satisfaction	0.415	***
Material satisfaction←Management of organization	0.415	***	Physiological state ←Organizational management	1.000	
Professional accomplishments←Safety psychology	0.261	***	Self-efficacy←Organizational management	1.452	***
Safety atmosphere←Safety psychology	−0.490	***	Professional degrees←Organizational management	0.559	***
Safety atmosphere←Material satisfaction	1.452	***	Responsibility consciousness←Non-skill internal cause	1.000	
Professional accomplishments←Non-skill internal cause	−0.108	***	Safety awareness←Non-skill internal cause	−0.108	***
Emotional attribution←Safety psychology	1.449	***	Group relationships←Non-skill internal cause	−2.843	***
Emotional attribution←Safety atmosphere	0.559	***	Emotional attribution←Professional accomplishments	1.000	
Career identity←Emotional attribution	1.000		Emotional ownership←Professional accomplishments	4.145	***
Career identity←Emotional attribution	1.476	***	Emotional ownership←Professional accomplishments	4.865	***
Career identity←Emotional attribution	0.732	***	Emotional attribution←Safety atmosphere	0.760	***
Emotional attribution←Safety atmosphere	1.000		Emotional attribution←Safety atmosphere	2.927	0.005

*** Note: *P < 0.1, **P < 0.05, P < 0.01.

## Conclusion

**Figure 6:**
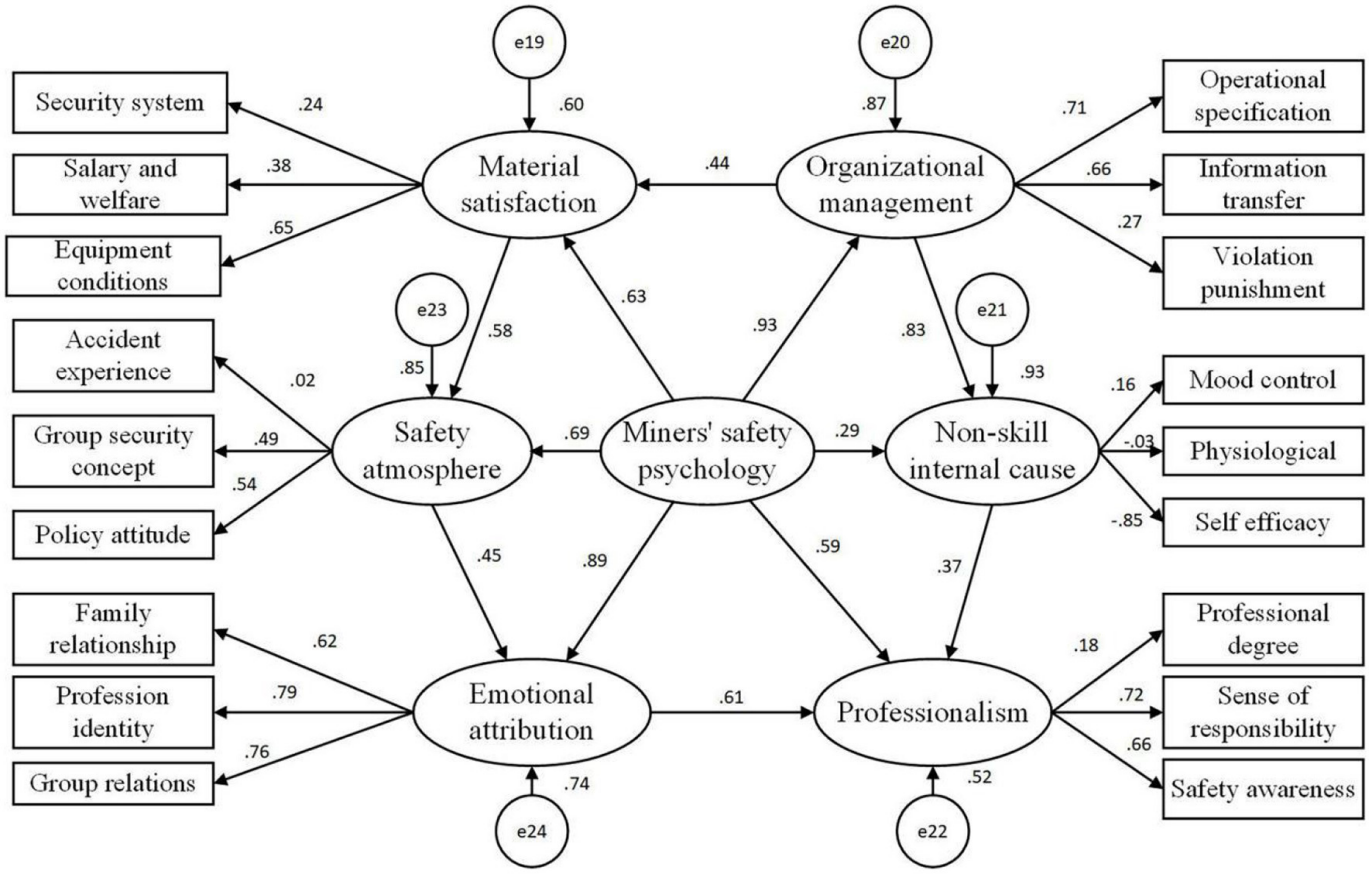
Path map of standardized coefficients of model.

By observing the load distribution data of 6 potential variables and 18 observation variables in the comprehensive model (Figure 6), we saw the following: in the new era, with improvements in living standards and production equipment, miners' safety psychology has been affected by the interaction of the self and environment and various potential variables. The structure of the safety demands of coal miners has undergone significant changes. The degree of influence and influence of each factor are different, and the correlations between each factor are also different. All of these facts create new challenges related to the actual needs of coal mine safety production management.

Emotional attribution was the most significant factor affecting the safety psychology of miners in the new era (influencing factor: 0.93). This shows that with the continuous improvements in miners' living standards, their spiritual needs instead of their material needs have begun to occupy the primary position in the miners' psychological needs, which coincides with Maslow's hierarchy of needs theory. At the same time, family relationships (influencing factor: 0.62), professional identity (influencing factor: 0.79), and group relationships (influencing factor: 0.76) had important effects on emotional attribution. Therefore, the effective ways to improve miners' safety psychology are to create a harmonious family atmosphere, improve miners' social status and social recognition, strengthen trade unions, and develop a rich and colorful collective life for workers. As pointed out in H3 and H4, there was a positive correlation between occupational literacy and safety atmosphere (the impact factors were 0.61 and 0.45, respectively). This shows that the miners' professional awareness, responsibility awareness, and safety operational ability affected their personal social positioning and work recognition, and this kind of personal emotional recognition was combined with the corresponding safety policies and organizational behavior, which together had an impact on miners' safety psychology.

The influence of organizational management was slightly lower than that of emotional attribution (influence factor: 0.89), which was still one of the most significant factors affecting miners' safety psychology in the new era. Organizational management was greatly influenced by organizational information transmission (influence factor: 0.66) and operational specifications (influence factor: 0.71). This shows that, in the new era, with the satisfaction of the material life of miners, an organizational consciousness begins to awaken, and they are willing to actively obtain organizational information and actively request standardized management of the organization, hope to be treated fairly, and care about and show willingness to participate in organizational life. At the same time, as a response to H1 and H6, organizational management had a positive impact on other important factors affecting miners' psychological safety, i.e., material satisfaction and non-skill endogenous factors (the impact factors were 0.44 and 0.83, respectively). This shows that perfect organizational management can alleviate the material needs of miners to a certain extent, and more importantly, perfect organizational management can effectively regulate the physiological functions, self-efficacy, and negative emotions of employees, thus having a positive impact on miners' safety psychology. Occupational literacy, material satisfaction, and safety atmosphere had a larger impact on miners' safety psychology (the influencing factors were 0.59, 0.63, and 0.69, respectively). As far as occupational literacy is concerned, miners' sense of responsibility and safety played a leading role in the formation of occupational literacy (the influencing factors were 0.72 and 0.66, respectively). At the same time, professional quality was related to the working mood, physiological state, and self-efficacy of miners. Although the salary level, welfare, and equipment conditions of miners have been greatly improved in the new era, they still play an important role in ensuring miners' psychological safety, which requires long-term attention from managers. As a response to H2, the formation of a safe atmosphere depends on stable material expectations, good group safety concepts, and miners' deep understanding of safety policies, which have a direct and important impact on miners' safety psychology. Surprisingly, the impact of non-skill on miners' safety psychology was at a low level (impact factor: 0.29), which differs from the previous literature. This may be related to the long-term work of miners in high-pressure and dangerous environments, which makes them develop highly stable emotional control and a certain degree of physiological adaptability. However, self-efficacy still had a large impact on non-skill internal factors (impact factor: 0.85). As mentioned in H5, the active self-efficacy construction of miners in organizational management can improve their professional accomplishments and promote the formation of miners' safety psychology.

Based on the actual needs of the current coal mining industry in China, and on the basis of a literature review, this paper constructed a comprehensive psychological evaluation model of miners' safety from the perspective of cognitive psychology, and uncovered the main and secondary factors affecting miners' safety psychology, as well as the influence of the interaction between them, in order to solve the “human error” problem in coal safety production. At the same time, considering that the choice of model path had a direct impact on the weight setting of the evaluation model and the choice of model path was not unique, the comprehensive model constructed in this study has better explanatory ability in some aspects. However, due to the study area, scope and sample size, there may be some inadequate explanations of some issues. Future research is necessary to further expand the sample size to study the safety psychology of coal miners in more coal mining enterprises in different regions, and needs to dedicate more time and manpower to building a more detailed comprehensive evaluation model, which can cover more aspects of the problem.

## Data Availability Statement

The original contributions presented in the study are included in the article/supplementary material, further inquiries can be directed to the corresponding author/s.

## Author Contributions

JZ contributed to the analysis and interpretation of data for the study. XW wrote the first draft of the manuscript. LY and JZ designed the framework for this study. ZQ, JG, and XZ contributed to the acquisition of data for this study. All authors approved the final manuscript.

## Funding

This study was supported by the National Natural Science Foundation of China (Nos. 71971003, 7210041523, and 72104001), the Major of National Social Science Foundation of China (20ZDA084), the Program of Humanities and Social Sciences in Colleges and Universities of Anhui Province, China (Nos. SK2020A0209 and SK2020A0210).

## Conflict of Interest

The authors declare that the research was conducted in the absence of any commercial or financial relationships that could be construed as a potential conflict of interest.

## Publisher's Note

All claims expressed in this article are solely those of the authors and do not necessarily represent those of their affiliated organizations, or those of the publisher, the editors and the reviewers. Any product that may be evaluated in this article, or claim that may be made by its manufacturer, is not guaranteed or endorsed by the publisher.
